# (2*E*,4*E*)-Ethyl 5-(phenyl­sulfon­yl)penta-2,4-dienoate

**DOI:** 10.1107/S1600536812009907

**Published:** 2012-03-17

**Authors:** Ulaganathan Sankar, V. Sabari, G. Suresh, Ramakrishnan Uma, S. Aravindhan

**Affiliations:** a Department of Chemistry, Pachaiyappa’s College, Chennai 600 030, India; b Department of Physics, Presidency College, Chennai 600 005, India

## Abstract

In the title compound, C_13_H_14_O_4_S, both C=C double bonds adopt an *E* conformation. In the crystal, mol­ecules are linked into centrosymmetric *R*_2_^2^(14) dimers *via* pairs of C—H⋯O hydrogen bonds.

## Related literature

For the biological activity of phenyl sulfonyl-containing compounds see: De-Benedetti *et al.* (1985 ▶). For a related structure, see: Li (2011 ▶). 
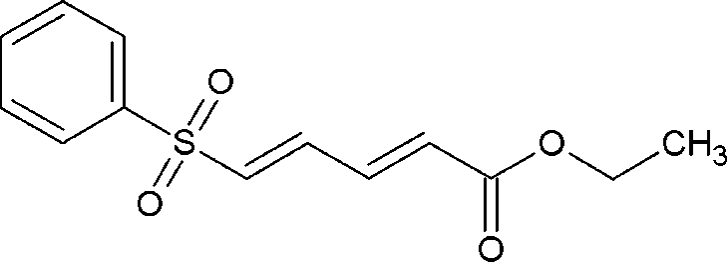


## Experimental

### 

#### Crystal data

C_13_H_14_O_4_S
*M* *_r_* = 266.30
Triclinic, 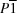

*a* = 6.2525 (3) Å
*b* = 7.8889 (4) Å
*c* = 14.5049 (7) Å
α = 82.828 (3)°
β = 87.261 (2)°
γ = 72.426 (2)°
*V* = 676.69 (6) Å^3^
*Z* = 2
Mo *K*α radiation
μ = 0.24 mm^−1^
*T* = 298 K
0.45 × 0.38 × 0.15 mm

#### Data collection

Bruker APEXII KappaCCD diffractometer
Absorption correction: multi-scan (*SADABS*; Bruker, 2008 ▶) *T*_min_ = 0.899, *T*_max_ = 0.965
8948 measured reflections
3084 independent reflections
2621 reflections with *I* > 2σ(*I*)
*R*_int_ = 0.020

#### Refinement

*R*[*F* ^2^ > 2σ(*F* ^2^)] = 0.043
*wR*(*F* ^2^) = 0.147
*S* = 1.87
3084 reflections
164 parameters
H-atom parameters constrained
Δρ_max_ = 0.29 e Å^−3^
Δρ_min_ = −0.30 e Å^−3^

### 

Data collection: *APEX2* (Bruker, 2008 ▶); cell refinement: *SAINT* (Bruker, 2008 ▶); data reduction: *SAINT*; program(s) used to solve structure: *SHELXS97* (Sheldrick, 2008 ▶); program(s) used to refine structure: *SHELXL97* (Sheldrick, 2008 ▶); molecular graphics: *ORTEP-3* (Farrugia, 1997 ▶); software used to prepare material for publication: *SHELXL97* and *PLATON* (Spek, 2009 ▶).

## Supplementary Material

Crystal structure: contains datablock(s) I, global. DOI: 10.1107/S1600536812009907/bt5831sup1.cif

Structure factors: contains datablock(s) I. DOI: 10.1107/S1600536812009907/bt5831Isup2.hkl

Supplementary material file. DOI: 10.1107/S1600536812009907/bt5831Isup3.cml

Additional supplementary materials:  crystallographic information; 3D view; checkCIF report

## Figures and Tables

**Table 1:** Hydrogen-bond geometry (Å, °)

*D*—H⋯*A*	*D*—H	H⋯*A*	*D*⋯*A*	*D*—H⋯*A*
C7—H7⋯O3^i^	0.93	2.32	3.212 (2)	161

Symmetry code: (i) 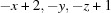.

**Fig. 1.**
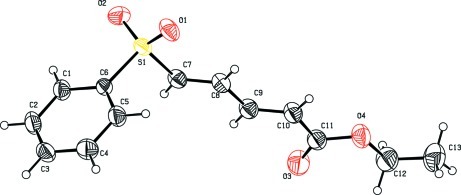
The molecular structure of the title compound with the atom numbering scheme. Displacement ellipsoids are shown at 30% probability level. The H atoms are presented as a small spheres of arbitrary radius. Related atoms have symmetry code: (i) -x + 2, -y, -z + 1.

**Fig. 2.**
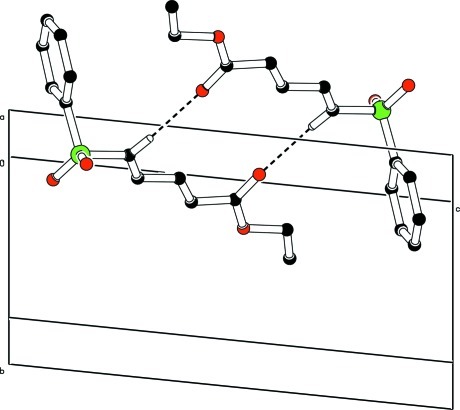
A view of the crystal packing. H atoms not involved in hydrogen bonding(dashed lines)have been omitted for clarity.

## supplementary crystallographic information

### Comment

Phenyl sulfonyl containing compounds show a wide range of biological properties (De-Benedetti *et al.*, 1985).

Fig. 1. shows a displacement ellipsoid plot of the title compound. Both C=C double bonds display an E configuration. The title molecule exhibits structural similarities with the already reported related structure (Li, 2011). The dihedral angle between two planes (C5—C6—S1—O1) and (C1—C6—S1—O2) is 37.32 (6)°. The crystal packing is stabilized by C—H···O intermolecular interactions. The molecules are linked into centrosymmetric *R*_2_^2^(14) dimers via C7—H7···O3 hydrogen bonds (Table 1). The packing of the compound is shown in (Fig. 2).

### Experimental

LIHMDS (8 ml, 8.4 mmol, 1.06 molar solution in THF) was added drop wise to a 0 °C cooled solution of bisphenyl sulfonyl methane (1 g, 3.4 mmol) in dried THF (15 ml) under argon atmosphere. The reaction mixture was stirred at same temp for 1 h, and then trans ethyl 4-bromo coronate (0.71 g, 3.7 mmol) in dry THF (5 ml) was added dropwise and the reaction mixture was allowed to come to RT and it was stirred under argon atmosphere for 16 h. The reaction mixture was quenched by adding saturated NH_4_Cl (20 ml) and then extracted with ethyl acetate (2x20 ml) and washed with water (2x20 ml) and sat brine (20 ml). Then, the organic layer was dried over MgSO_4_. Evaporation of the solvent under vacuum furnished the crude product, the residue was chromatographed (25% ethyl acetate in hexanes) to give analytically pure (2E, 4E)-ethyl 5-(phenyl sulfonyl)penta-2,4-dienote (0.673 g. yield 75%) as a colorless solid.

### Refinement

Hydrogen atoms were placed in calculated positions with C–H ranging from 0.93 Å to 0.97Å and refined using a the riding model with fixed isotropic displacement parameters: *U*_iso_(H) = 1.5 *U*_eq_(C) for the methyl group and *U*_iso_(H) = 1.2 *U*_eq_(C) for other groups.

### Crystal data

**Table:**

C_13_H_14_O_4_S	*Z* = 2
*M**_r_* = 266.30	*F*(000) = 280
Triclinic, *P*1	*D*_x_ = 1.307 Mg m^−^^3^
*a* = 6.2525 (3) Å	Mo *K*α radiation, λ = 0.71073 Å
*b* = 7.8889 (4) Å	Cell parameters from 5946 reflections
*c* = 14.5049 (7) Å	θ = 2.7–28.3°
α = 82.828 (3)°	µ = 0.24 mm^−^^1^
β = 87.261 (2)°	*T* = 298 K
γ = 72.426 (2)°	Triclinic, colourless
*V* = 676.69 (6) Å^3^	0.45 × 0.38 × 0.15 mm

### Data collection

**Table:**

Bruker APEXII KappaCCD diffractometer	3084 independent reflections
Radiation source: fine-focus sealed tube	2621 reflections with *I* > 2σ(*I*)
Graphite monochromator	*R*_int_ = 0.020
Detector resolution: 15.9948 pixels mm^-1^	θ_max_ = 28.4°, θ_min_ = 2.7°
ω and φ scans	*h* = −8→8
Absorption correction: multi-scan (*SADABS*; Bruker, 2008)	*k* = −10→9
*T*_min_ = 0.899, *T*_max_ = 0.965	*l* = −19→19
8948 measured reflections	

### Refinement

**Table:**

Refinement on *F*^2^	Secondary atom site location: difference Fourier map
Least-squares matrix: full	Hydrogen site location: inferred from neighbouring sites
*R*[*F*^2^ > 2σ(*F*^2^)] = 0.043	H-atom parameters constrained
*wR*(*F*^2^) = 0.147	*w* = 1/[σ^2^(*F*_o_^2^) + (0.0551*P*)^2^] where *P* = (*F*_o_^2^ + 2*F*_c_^2^)/3
*S* = 1.87	(Δ/σ)_max_ = 0.001
3084 reflections	Δρ_max_ = 0.29 e Å^−^^3^
164 parameters	Δρ_min_ = −0.30 e Å^−^^3^
0 restraints	Extinction correction: *SHELXL97* (Sheldrick, 2008)
Primary atom site location: structure-invariant direct methods	Extinction coefficient: 0.0173 (18)

### Special details

**Table:**

Geometry. All e.s.d.'s (except the e.s.d. in the dihedral angle between two l.s. planes) are estimated using the full covariance matrix. The cell e.s.d.'s are taken into account individually in the estimation of e.s.d.'s in distances, angles and torsion angles; correlations between e.s.d.'s in cell parameters are only used when they are defined by crystal symmetry. An approximate (isotropic) treatment of cell e.s.d.'s is used for estimating e.s.d.'s involving l.s. planes.
Refinement. Refinement of *F*^2^ against ALL reflections. The weighted *R*-factor *wR* and goodness of fit *S* are based on *F*^2^, conventional *R*-factors *R* are based on *F*, with *F* set to zero for negative *F*^2^. The threshold expression of *F*^2^ > σ(*F*^2^) is used only for calculating *R*-factors(gt) *etc*. and is not relevant to the choice of reflections for refinement. *R*-factors based on *F*^2^ are statistically about twice as large as those based on *F*, and *R*- factors based on ALL data will be even larger.

### Fractional atomic coordinates and isotropic or equivalent isotropic displacement parameters (Å^2^)

**Table:**

	*x*	*y*	*z*	*U*_iso_*/*U*_eq_	
C1	1.5671 (3)	−0.0639 (2)	0.13787 (12)	0.0577 (4)	
H1	1.6549	−0.0001	0.1586	0.069*	
C2	1.6638 (3)	−0.2359 (3)	0.11608 (12)	0.0670 (5)	
H2	1.8179	−0.2881	0.1219	0.080*	
C3	1.5361 (3)	−0.3300 (2)	0.08618 (12)	0.0634 (5)	
H3	1.6035	−0.4465	0.0726	0.076*	
C4	1.3103 (3)	−0.2555 (3)	0.07586 (14)	0.0714 (5)	
H4	1.2246	−0.3206	0.0549	0.086*	
C5	1.2088 (3)	−0.0820 (2)	0.09680 (12)	0.0612 (4)	
H5	1.0550	−0.0299	0.0895	0.073*	
C6	1.3387 (2)	0.0124 (2)	0.12854 (9)	0.0446 (3)	
C7	1.0978 (3)	0.1812 (2)	0.27041 (10)	0.0534 (4)	
H7	1.1952	0.1132	0.3169	0.064*	
C8	0.8815 (3)	0.2378 (2)	0.29034 (11)	0.0545 (4)	
H8	0.7812	0.3023	0.2441	0.065*	
C9	0.7980 (3)	0.2008 (2)	0.38347 (11)	0.0585 (4)	
H9	0.9013	0.1296	0.4271	0.070*	
C10	0.5861 (3)	0.2604 (2)	0.41125 (11)	0.0603 (4)	
H10	0.4768	0.3307	0.3697	0.072*	
C11	0.5253 (3)	0.2140 (3)	0.50916 (12)	0.0616 (4)	
C12	0.2457 (4)	0.2713 (3)	0.62613 (15)	0.0902 (7)	
H12A	0.2830	0.1440	0.6466	0.108*	
H12B	0.3229	0.3242	0.6655	0.108*	
C13	0.0051 (4)	0.3538 (4)	0.63268 (17)	0.1022 (8)	
H13A	−0.0313	0.4779	0.6084	0.153*	
H13B	−0.0420	0.3436	0.6966	0.153*	
H13C	−0.0704	0.2944	0.5974	0.153*	
O1	1.0292 (2)	0.32081 (16)	0.09966 (8)	0.0719 (4)	
O2	1.3810 (2)	0.30846 (17)	0.17469 (9)	0.0749 (4)	
O3	0.6500 (2)	0.1095 (2)	0.56403 (9)	0.0889 (5)	
O4	0.3154 (2)	0.3006 (2)	0.52876 (8)	0.0778 (4)	
S1	1.20983 (6)	0.22766 (5)	0.16117 (3)	0.05254 (19)	

### Atomic displacement parameters (Å^2^)

**Table:**

	*U*^11^	*U*^22^	*U*^33^	*U*^12^	*U*^13^	*U*^23^
C1	0.0426 (8)	0.0583 (10)	0.0721 (10)	−0.0123 (7)	0.0000 (7)	−0.0144 (8)
C2	0.0466 (9)	0.0661 (12)	0.0762 (11)	0.0015 (8)	0.0056 (8)	−0.0106 (9)
C3	0.0706 (11)	0.0474 (9)	0.0635 (9)	−0.0046 (8)	0.0109 (8)	−0.0111 (7)
C4	0.0729 (12)	0.0618 (12)	0.0873 (12)	−0.0260 (10)	0.0008 (10)	−0.0236 (10)
C5	0.0462 (8)	0.0578 (10)	0.0782 (11)	−0.0109 (8)	−0.0021 (8)	−0.0140 (8)
C6	0.0428 (7)	0.0419 (8)	0.0454 (7)	−0.0087 (6)	0.0009 (6)	−0.0020 (6)
C7	0.0576 (9)	0.0475 (9)	0.0494 (8)	−0.0075 (7)	−0.0029 (7)	−0.0041 (6)
C8	0.0565 (9)	0.0496 (9)	0.0516 (8)	−0.0064 (7)	−0.0025 (7)	−0.0074 (7)
C9	0.0590 (10)	0.0562 (10)	0.0548 (9)	−0.0099 (8)	−0.0007 (7)	−0.0047 (7)
C10	0.0572 (10)	0.0603 (11)	0.0552 (9)	−0.0060 (8)	−0.0003 (7)	−0.0046 (8)
C11	0.0595 (10)	0.0586 (11)	0.0615 (10)	−0.0110 (8)	0.0010 (8)	−0.0047 (8)
C12	0.0948 (15)	0.0925 (16)	0.0674 (11)	−0.0119 (13)	0.0204 (11)	0.0019 (11)
C13	0.0908 (16)	0.122 (2)	0.0886 (15)	−0.0233 (15)	0.0273 (13)	−0.0240 (15)
O1	0.0726 (8)	0.0597 (8)	0.0600 (7)	0.0108 (6)	−0.0020 (6)	0.0051 (5)
O2	0.0824 (9)	0.0565 (8)	0.0942 (9)	−0.0331 (7)	0.0119 (7)	−0.0141 (7)
O3	0.0740 (9)	0.1009 (12)	0.0691 (8)	−0.0048 (8)	−0.0020 (7)	0.0225 (7)
O4	0.0705 (8)	0.0792 (10)	0.0640 (7)	0.0013 (7)	0.0155 (6)	0.0001 (7)
S1	0.0557 (3)	0.0399 (3)	0.0553 (3)	−0.00635 (19)	0.00194 (18)	−0.00117 (17)

### Geometric parameters (Å, º)

**Table:**

C1—C2	1.378 (2)	C8—H8	0.9300
C1—C6	1.378 (2)	C9—C10	1.326 (2)
C1—H1	0.9300	C9—H9	0.9300
C2—C3	1.359 (2)	C10—C11	1.483 (2)
C2—H2	0.9300	C10—H10	0.9300
C3—C4	1.363 (3)	C11—O3	1.195 (2)
C3—H3	0.9300	C11—O4	1.319 (2)
C4—C5	1.389 (3)	C12—C13	1.451 (3)
C4—H4	0.9300	C12—O4	1.469 (2)
C5—C6	1.383 (2)	C12—H12A	0.9700
C5—H5	0.9300	C12—H12B	0.9700
C6—S1	1.7595 (15)	C13—H13A	0.9600
C7—C8	1.320 (2)	C13—H13B	0.9600
C7—S1	1.7434 (15)	C13—H13C	0.9600
C7—H7	0.9300	O1—S1	1.4294 (12)
C8—C9	1.452 (2)	O2—S1	1.4328 (13)
			
C2—C1—C6	119.12 (15)	C8—C9—H9	117.4
C2—C1—H1	120.4	C9—C10—C11	119.36 (17)
C6—C1—H1	120.4	C9—C10—H10	120.3
C3—C2—C1	120.67 (15)	C11—C10—H10	120.3
C3—C2—H2	119.7	O3—C11—O4	123.62 (16)
C1—C2—H2	119.7	O3—C11—C10	124.36 (17)
C2—C3—C4	120.76 (17)	O4—C11—C10	112.01 (15)
C2—C3—H3	119.6	C13—C12—O4	108.24 (19)
C4—C3—H3	119.6	C13—C12—H12A	110.1
C3—C4—C5	119.78 (17)	O4—C12—H12A	110.1
C3—C4—H4	120.1	C13—C12—H12B	110.1
C5—C4—H4	120.1	O4—C12—H12B	110.1
C6—C5—C4	119.32 (16)	H12A—C12—H12B	108.4
C6—C5—H5	120.3	C12—C13—H13A	109.5
C4—C5—H5	120.3	C12—C13—H13B	109.5
C1—C6—C5	120.34 (15)	H13A—C13—H13B	109.5
C1—C6—S1	119.90 (12)	C12—C13—H13C	109.5
C5—C6—S1	119.71 (11)	H13A—C13—H13C	109.5
C8—C7—S1	123.19 (13)	H13B—C13—H13C	109.5
C8—C7—H7	118.4	C11—O4—C12	115.52 (15)
S1—C7—H7	118.4	O1—S1—O2	119.36 (8)
C7—C8—C9	120.95 (15)	O1—S1—C7	108.53 (8)
C7—C8—H8	119.5	O2—S1—C7	107.09 (8)
C9—C8—H8	119.5	O1—S1—C6	109.53 (7)
C10—C9—C8	125.22 (16)	O2—S1—C6	108.57 (7)
C10—C9—H9	117.4	C7—S1—C6	102.40 (7)
			
C6—C1—C2—C3	−0.3 (3)	O3—C11—O4—C12	4.5 (3)
C1—C2—C3—C4	0.9 (3)	C10—C11—O4—C12	−176.12 (16)
C2—C3—C4—C5	−0.5 (3)	C13—C12—O4—C11	−171.7 (2)
C3—C4—C5—C6	−0.4 (3)	C8—C7—S1—O1	−4.46 (18)
C2—C1—C6—C5	−0.7 (2)	C8—C7—S1—O2	125.64 (16)
C2—C1—C6—S1	176.98 (12)	C8—C7—S1—C6	−120.23 (16)
C4—C5—C6—C1	1.0 (2)	C1—C6—S1—O1	144.75 (14)
C4—C5—C6—S1	−176.61 (14)	C5—C6—S1—O1	−37.59 (14)
S1—C7—C8—C9	−177.71 (12)	C1—C6—S1—O2	12.83 (15)
C7—C8—C9—C10	176.02 (18)	C5—C6—S1—O2	−169.51 (12)
C8—C9—C10—C11	−179.12 (15)	C1—C6—S1—C7	−100.21 (14)
C9—C10—C11—O3	−8.3 (3)	C5—C6—S1—C7	77.46 (14)
C9—C10—C11—O4	172.26 (17)		

### Hydrogen-bond geometry (Å, º)

**Table:**

*D*—H···*A*	*D*—H	H···*A*	*D*···*A*	*D*—H···*A*
C7—H7···O3^i^	0.93	2.32	3.212 (2)	161

Symmetry code: (i) −*x*+2, −*y*, −*z*+1.
